# Extracellular volume quantification using synthetic haematocrit assessed from native and post-contrast longitudinal relaxation T1 times of a blood pool

**DOI:** 10.1186/s12872-021-02179-z

**Published:** 2021-07-31

**Authors:** Lukas Opatril, Roman Panovsky, Jan Machal, Tomas Holecek, Lucia Masarova, Vera Feitova, Vladimir Kincl, Marek Hodejovsky, Lenka Spinarova

**Affiliations:** 1 1st Department of Internal Medicine and Cardioangiology, St. Anne’s University Hospital, Brno, Czech Republic; 2 International Clinical Research Center, St. Anne’s University Hospital, Brno, Czech Republic; 3 Faculty of Medicine, Masaryk University, Brno, Czech Republic; 4 Department of Medical Imaging, St. Anne’s University Hospital, Brno, Czech Republic; 5 Department of Pathophysiology, Faculty of Medicine, Masaryk University, Brno, Czech Republic; 6 1st Department of Internal Medicine and Cardioangiology, International Clinical Research Center, St. Anne’s University Hospital, Brno, Czech Republic

**Keywords:** Extracellular volume, ECV, Synthetic haematocrit, Cardiovascular magnetic resonance, CMR

## Abstract

**Background:**

In terms of cardiovascular magnetic resonance are haematocrit values required for calculation of extracellular volume fraction (ECV). Previously published studies have hypothesized that haematocrit could be calculated from T1 blood pool relaxation time, however only native T1 relaxation time values have been used and the resulting formulae had been both in reciprocal and linear proportion. The aim of the study was to generate a synthetic haematocrit formula from only native relaxation time values first, calculate whether linear or reciprocal model is more precise in haematocrit estimation and then determine whether adding post-contrast values further improve its precision.

**Methods:**

One hundred thirty-nine subjects underwent CMR examination. Haematocrit was measured using standard laboratory methods. Afterwards T1 relaxation times before and after the application of a contrast agent were measured and a statistical relationship between these values was calculated.

**Results:**

Different linear and reciprocal models were created to estimate the value of synthetic haematocrit and ECV. The highest coefficient of determination was observed in the combined reciprocal model “− 0.047 + (779/ blood native) − (11.36/ blood post-contrast)”.

**Conclusions:**

This study provides more evidence that assessing synthetic haematocrit and synthetic ECV is feasible and statistically most accurate model to use is reciprocal. Adding post-contrast values to the calculation was proved to improve the precision of the formula statistically significantly.

## Introduction

Cardiovascular magnetic resonance (CMR) provides a lot of non-invasively acquired information about the myocardium. One of the sequences used is T1 mapping [1, 2], which allows us to measure myocardial extracellular volume (ECV), using T1 relaxation time values acquired before and after the administration of a gadolinium contrast agent. However, haematocrit (Htc) is related to the blood volume of distribution and is also required to calculate ECV. Higher ECV values have already proven to be a pathological finding in many cardiovascular diseases [2–12]. There are already studies showing a strong correlation between ECV measured by CMR and histological findings [13, 14].

Some authors have already published their findings and formulae in this field [3, 4, 15–18] and as some other studies have already shown—the T1 relaxation times obtained while using Modified Look-Locker Imaging (MOLLI) sequences still show statistically significant differences between different types of scanners with the same field strength (both 1.5T and 3T) [19, 20]. In addition, as Treibel stated in his study [17], further comparisons and potentially a local calibration in every single centre for synthetic Htc and ECV calculations are required. While some other authors have used Shortened Modified Look-Locker Imaging (Sh-MOLLI) or Sh-MOLLI and MOLLI sequences [15, 17], exclusively MOLLI sequences have been used in our study. Other studies have also used different formulae for calculating synthetic Htc—with mostly the reciprocal proportion used [3, 4, 16, 17].

Furthermore, we hypothesise that adding post-contrast T1 relaxation time of the blood pool and therefore more data to the calculation could improve the formula. To our knowledge, post-contrast values of a blood pool have never been added to the formula calculating synthetic Htc before.

This study could provide more evidence whether linear or reciprocal regression is to be used while calculating a synthetic Htc for synthetic ECV, if the formula obtained by using an Ingenia 1.5 T scanner resembles any other obtained with a different scanner and if a statistically relevant improvement can be achieved by adding post-contrast values to the calculation.

## Methods

The aim of the study was to generate a formula to determine a synthetic Htc and ECV from the T1 blood relaxation times using MOLLI sequences obtained with an Ingenia 1.5T scanner and to determine whether linear or reciprocal regression is statistically more relevant to our data. We aimed to find a correlation between Htc and T1 relaxation times firstly using only native values, and secondly considering both native and post-contrast values. Formulae created were used for ECV quantification and compared with values acquired using different formulae and laboratory methods.

This retrospective study included 139 subjects with a wide range of diagnoses, who underwent CMR examination using an Ingenia 1.5T scanner using both native and contrast methods. Inclusion criteria were both native and post-contrast T1 mapping sequences available and a blood sample collected right before the CMR examination (several minutes). The patients consisted of 5 groups following primary diagnoses: chronic obstructive pulmonary disease, Duchene muscular dystrophy female carriers, a group after anthracycline treatment, patients after a heart transplant, and controls. Control group included patients with a clinical indication for CMR examination, but normal CMR findings, other cardiac results and no other relevant medical history (Table 1). The appropriate size of the group to reveal even medium size effects was checked using the online available calculator for multiple regression [21], with parameters set as f^2^ = 0.15, power level (1 − β) = 0.8, α = 0.05, and 3 predictors. The number of patients enrolled well exceeded the necessary minimum of n = 76.

**Table 1:** Characteristics of the patient groups: genetic carriers of Duchenne muscular dystrophy, patients after anthracycline treatment, heart transplant, chronic obstructive pulmonary disease and controls

Primary diagnosis	Number of patients	Age	Female	Mean laboratory Htc	Mean T1 blood relaxation time—native	Mean T1 blood relaxation time—post-contrast
Overall	139	35.42 ± 15.97	76 (54.7%)	0.41 ± 0.04	1545.78 ± 84.24	229.76 ± 27.03
DMD carriers	40	37.93 ± 11.85	40 (100%)	0.4 ± 0.03	1560.33 ± 79.66	223.92 ± 24.64
Patients after anthracycline treatment	71	25.99 ± 5	26 (36.6%)	0.42 ± 0.04	1534 ± 80.12	230.9 ± 29.02
HT	8	53.63 ± 13.51	2 (25%)	0.4 ± 0.08	1563.5 ± 104.27	234 ± 26.46
COPD	15	67.33 ± 8.5	5 (33.3%)	0.42 ± 0.04	1566.13 ± 70.54	235.67 ± 19.72
Controls	5	24.4 ± 2.58	3 (60%)	0.44 ± 0.04	1496.8 ± 69.15	235.8 ± 24.21

Values represent the number of patients or the median ± standard deviation

Htc = haematocrit, DMD carriers = genetic carriers of Duchenne muscular dystrophy, HT = heart transplant, COPD = chronic obstructive pulmonary disease

Patients had their Htc measured using standard laboratory methods in a centralised hospital laboratory. Afterwards, the patients underwent CMR including T1 mapping using MOLLI sequences both before the administration of a contrast agent and 15 min after. Similarly as described previously [22]—a balanced single-shot T1-TFE sequence with an inversion prepulse, cardiac triggering and breath-hold technique in the mid-ventricular level in the short-axis was used. With a 5s (3s) 3s MOLLI scheme for native T1 and 4s (1s) 3s (1s) 2s for enhanced T1 mapping was used with typical imaging parameters as follows: FOV 300 × 300 mm, reconstruction matrix 256, slice thickness 10 mm, acquisition voxel size 2.00 × 2.00 × 10.00 mm, time to repetition (TR) ≈ 2.2 ms, echo time (TE) ≈ 1.1 ms, flip angle 35°, SENSE factor 2. For the contrast agent, gadolinium (Gadovist, Bayer AG, Leverkusen, Germany) in the dosage of 0.2 mmol/kg was used.

**Fig. 1:**
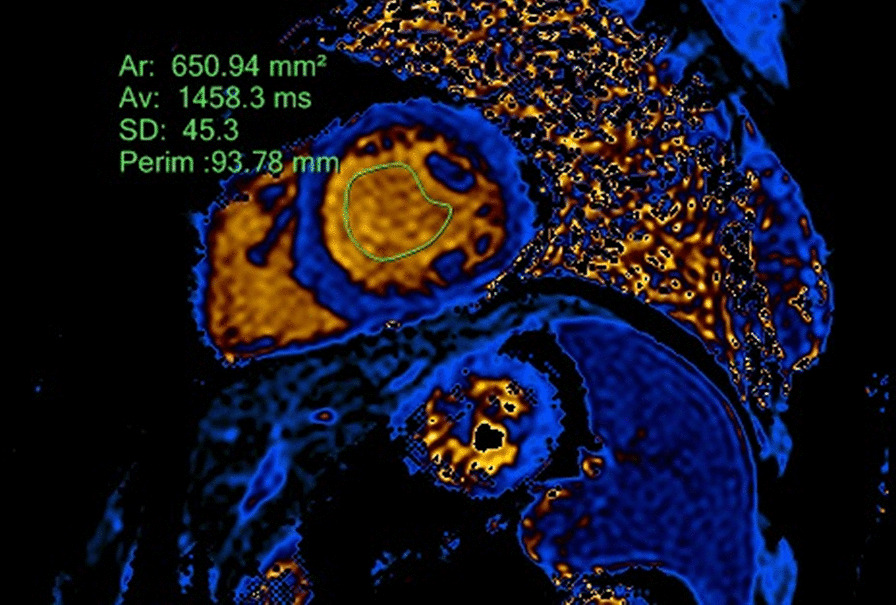
Measurement of T1 blood relaxation time in a native T1 mapping image

**Fig. 2:**
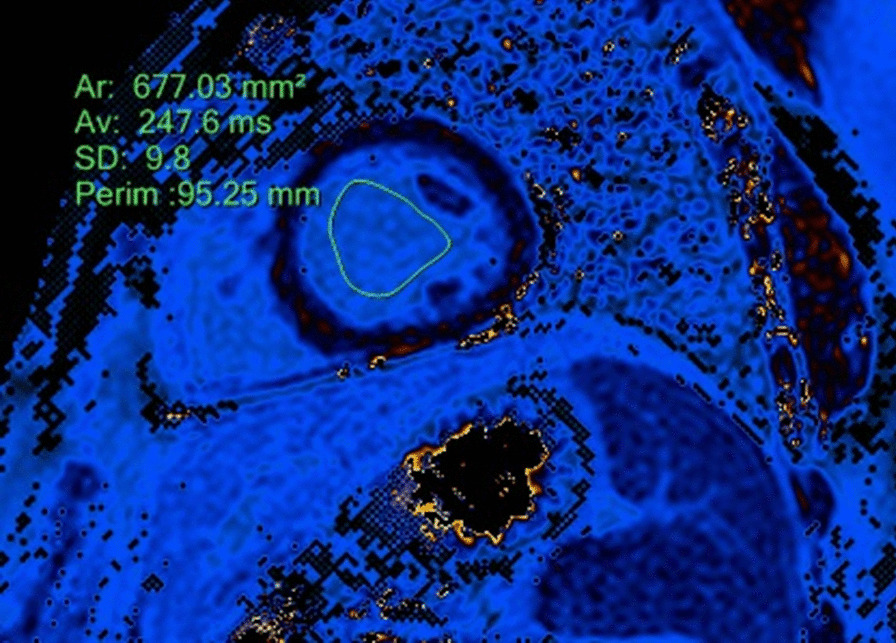
Measurement of T1 blood relaxation time in a postcontrast T1 mapping image

The regions of interest (ROIs) were contoured in both native and post-contrast images acquired 15 min after the contrast injection, including only a blood pool without any papillary muscles (Figs. 1, 2). First, the formula and correlation coefficient using only native values of the blood pool was calculated; afterwards, the same calculation was performed using both native and post-contrast values. Acquired Htc values were used to quantify ECV.

As for statistical analysis—Pearson correlation coefficients and related p-values were computed to assess the correlation between Htc and CMR-derived blood native and blood enhanced values. Four regression models to estimate Htc from blood native and blood post-contrast values were created in the following fashion:

1. (native linear): Htc_est_ = b + (T_1-BN_/a)
2. (native reciprocal): Htc_est_ = b + (a/T_1-BN_)
3. (combined linear): Htc_est_ = c + (T_1-BN_/a) + (T_1-BP_/b)
4. (combined reciprocal): Htc_est_ = c + (a/T_1-BN_) + (b/T_1-BP_)

Where Htc_est_ is an estimated value of haematocrit, a, b, c are constants, T_1-BN_ is a blood native value and T_1-BP_ is a blood post-contrast (enhanced) value.

Further, using the estimated or measured Htc value, the extracellular volume was calculated as:

$${\text{ECV}} = \left( {1{-}{\text{Htc}}} \right)*\left( {\left( {\left( {1/{\text{T}}_{{\text{1 - MP}}} } \right){-}\left( {1/{\text{T}}_{{\text{1 - MN}}} } \right)} \right)/\left( {\left( {1/{\text{T}}_{{\text{1 - BP}}} } \right){-}\left( {1/{\text{T}}_{{\text{1 - BN}}} } \right)} \right)} \right)$$

where T_1-MN_ is a myocardium native value and T_1-MP_ is a myocardium post-contrast value. The ECV calculated using either measured or estimated Htc was compared to the values based on linear and reciprocal models by Treibel et al. using blood native values obtained by MOLLI and ShMOLLI [15, 17] and Bland–Altman analysis was performed to assess the systematic bias.

The statistical significance of these factors was assessed. To compare the linear and reciprocal models, a coefficient of determination (r^2^) was attributed to each model. A residual analysis was employed to confirm the adequacy of each model for the estimation of Htc and the residual variance of the model with the highest r^2^ was compared with other models using an F-test.

Finally, as the study group was heterogeneous and consisted of patients suffering from several diagnoses, the possible effect of the primary diagnosis was determined using an Analysis of Covariance (ANCOVA), with the primary diagnosis as an independent factor in the model.

Normality was tested by Kolmogorov–Smirnov test of normality and by visual inspection of histograms. To exclude substantial multicollinearity between the variables used in a model, the variance inflation factor (VIF) was computed for a model containing blood native value as an independent and blood post-contrast value as a dependent variable, as well as for the diagnoses (nominal data, one binary variable per each diagnosis, independent) and the blood native / blood post contrast value (dependent variable). Value > 2.5 was considered as a substantial multicollinearity.

Generally, results with P < 0.05 were regarded as statistically significant. The analysis was performed using STATISTICA 13.2 (TIBCO software, The United States of America).

## Results

**Fig. 3:**
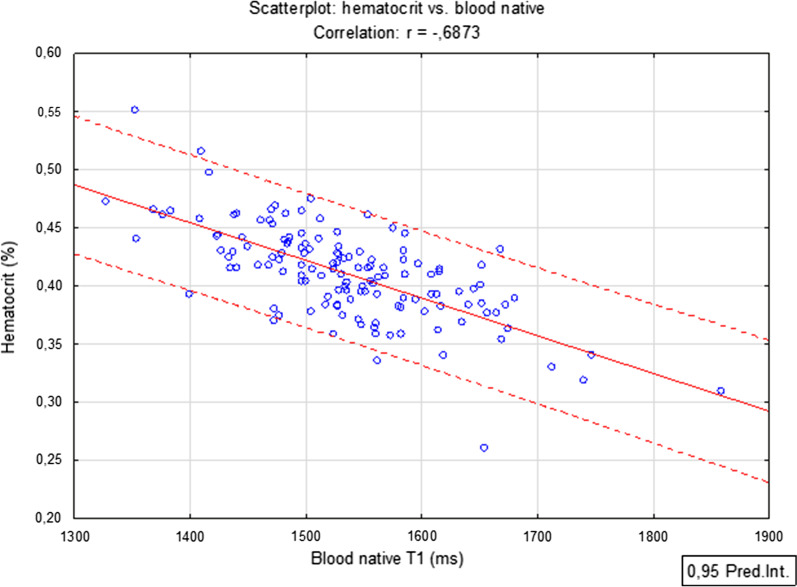
Scatter plot showing the correlation between laboratory measured Htc and native T1 blood relaxation time

**Fig. 4:**
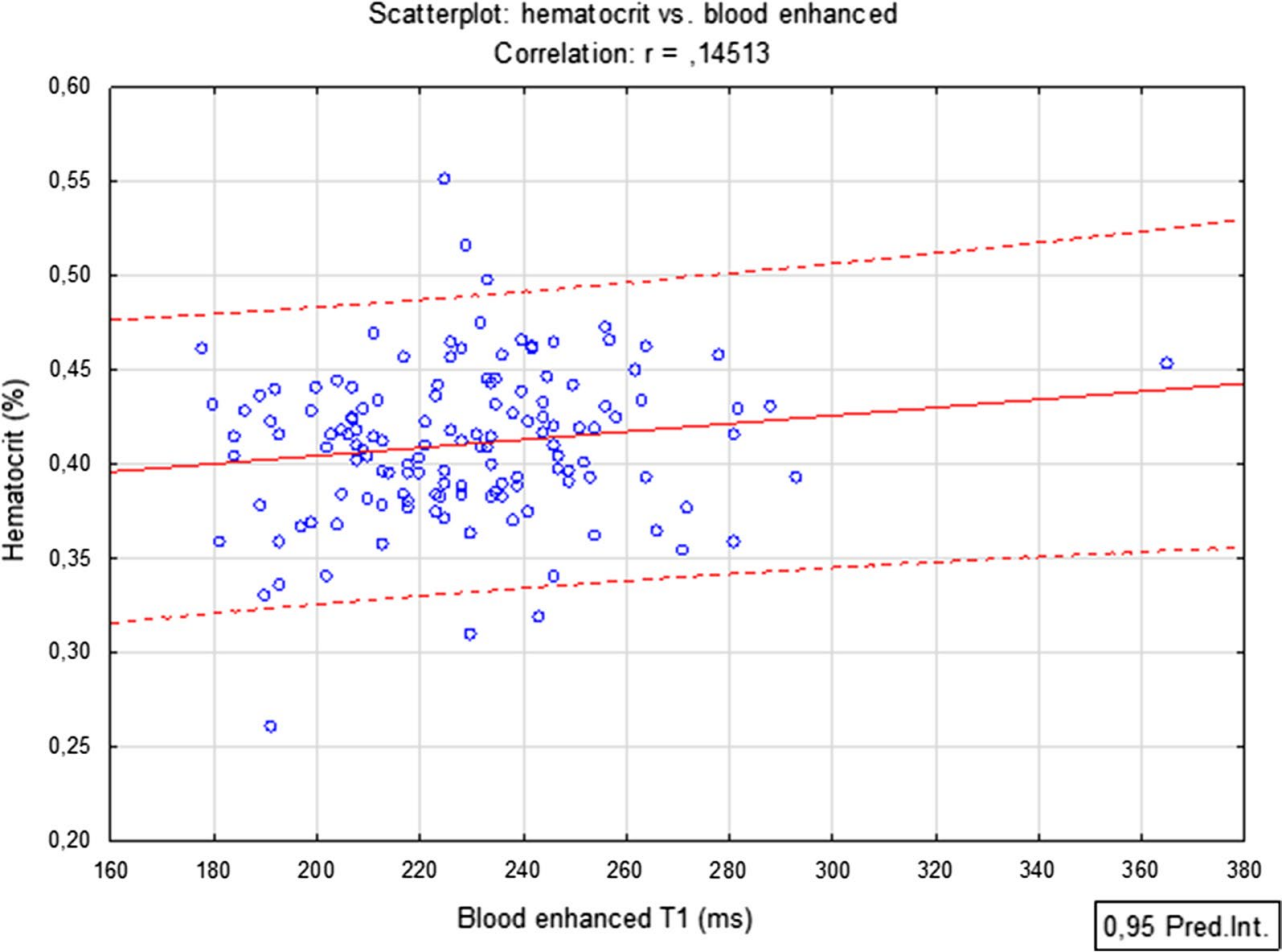
Scatter plot showing the correlation between laboratory measured Htc and post-contrast T1 blood relaxation time

The blood native value showed a moderately strong negative correlation with Htc, (r = − 0.68, P < 0.001) (Fig. 3), while using only the blood post-contrast value showed only a mildly positive correlation, which lacked statistical significance (r = 0.15, P = 0.09) (Fig. 4). These two CMR-derived parameters were not correlated between themselves (r = − 0.02, P = 0.81). The value of VIF was very small (1.0004), effectively excluding any collinearity. The parameters were thus regarded as independent. The p-value of Kolmogorov–Smirnov test was > 0.10 for all measured parameters in CMR, as well as for Htc value, and the histograms corresponded with the Gaussian distribution in all cases.

Using native and post-contrast blood pool values, the following formulae were created:

1. “Htc_est_ = 0.914 − (T_1-BN_/3051)”, where the blood native value significantly contributed to the estimation (P < 0.001)
2. “Htc_est_ = -0.098 + (779/T_1-BN_)”, where the blood native value significantly contributed to the estimation (P < 0.001)
3. “Htc_est_ = 0.870 − (T_1-BN_/3061) + (blood post-contrast T_1-BP_/5392)”, where both factors significantly contributed to the Htc estimation (T_1-BN_: P < 0.001, T_1-BP_: P = 0.043).
4. “Htc_est_ = -0.047 + (779/(T_1-BN_)) − (11.36/T_1-BP_)”, where both factors significantly contributed to the Htc estimation (T_1-BN_: P < 0.001, T_1-BP_: P = 0.022).

The highest coefficient of determination (0.49) was observed in the combined reciprocal model (4), followed by 0.48 for the combined linear model (3) and 0.47 for both models using native values only (models 2 and 1). Correspondingly, the variance of residuals decreased from model 1 to model 4, as there was no statistically significant difference in the variance of residuals (P > 0.05 in all cases).

The descriptive statistics of ECV values obtained by different methods including the Treibel et al. reciprocal (Htc = 866 * (1/T_1-BN_) − 0.1232) [17] model used in several different articles and other linear models, is shown in Table 2.

**Table 2:** ECV values obtained by different methods of haematocrit estimation

Method of haematocrit estimation	ECV value	ECV bias (95% CI) [%]*	Correlation mean vs. bias (r)	Correlation mean vs. bias (p-value)	r^2^ in haematocrit estimation*
Direct measurement	0.250 ± 0.035	–	–	–	–
Native linear (model 1)	0.250 ± 0.035	0.2 (− 0.7 to 1.1) %	0.03	0.74	0.47
Native reciprocal (model 2)	0.250 ± 0.035	0.2 (− 0.7 to 1.1) %	0.03	0.73	0.47
Combined linear (model 3)	0.250 ± 0.034	0.2 (− 0.7 to 1.0) %	− 0.02	0.78	0.48
Combined reciprocal (model 4)	0.250 ± 0.034	0.2 (− 0.7 to 1.0) %	− 0.03	0.73	0.49
Treibel et al. linear MOLLI**	0.238 ± 0.033	− 4.6% (− 5.5 to − 3.8) %	− 0.13	0.13	0.47
Treibel et al. reciprocal MOLLI**	0.237 ± 0.034	− 5.2 (− 6.0 to − 4.3) %	− 0.03	0.74	0.47
Treibel et al. reciprocal shMOLLI	0.252 ± 0.035	0.8 (− 0.1 to 1.6) %	0.01	0.93	0.47

^*^Compared to the direct measurement of haematocrit value

^**^Significant bias compared to values obtained by direct measurement of Htc

CI = confidence interval, ECV = extracellular volume, MOLLI = Modified Look-Locker Imaging, Sh-MOLLI = Shortened Modified Look-Locker Imaging

Interestingly, when compared with Treibel’s formulae, there was a significant bias in the case of MOLLI-based empirical formulae that rendered significantly lower results with around 5% bias in both cases, while there was no significant bias in the case of ShMOLLi-based formula (bias < 1%; the 95% confidence interval involved zero). The bias was independent on the value of ECV in all cases (p for trend > 0.05). As expected, the models based on our data had negligible bias close to 0.

Finally, the primary diagnosis was added to the models as an independent factor. The multicollinearity of primary diagnosis and either blood native (VIF = 1.09) or blood post-contrast (VIF = 1.03) T1 values was negligible, confirming the appropriateness of the model. In neither case did the factor of primary diagnosis contribute to the Htc estimation (P > 0.05 in all cases). The age itself did not correlate with Htc either (Spearman r = 0.05, p = 0.59). The formulae were thus applicable to the whole study population, regardless of the primary diagnosis status and age.

## Discussion

This study provides more information about synthetic Htc calculation from the T1 relaxation times of a blood pool acquired using T1 mapping sequences on 1.5 T Ingenia scanner. Adding post-contrast values to the formula showed a mild, but statistically relevant improvement of the synthetic Htc calculation. The frequency of using synthetic ECV maps and thus calculating synthetic Htc values is increasing. This study brings evidence that by simply adding post-contrast values to the calculation formula a statistically relevant improvement of the assessment accuracy could be achieved and also implies that results assessed using reciprocal regression to T1 relaxation times of blood pool are statistically more accurate.

In other studies, most authors have used reciprocal regression to T1 relaxation times [3, 17], several others [4, 16] have used the formula from Treibel’s 2016 article [17]. Only Treibel in his 2015 study [15] has used linear regression to T1 blood relaxation times. Previous studies have used only native T1 relaxation times, whereas in this study a post-contrast T1 relaxation times were added to the formulae, resulting in a mild, but statistically relevant improvement of the assessed synthetic Htc and therefore synthetic ECV accuracy.

This study was a single centre, which means there is a certain selection bias, however, this ensures similarity of the protocol, sequences and magnetic resonance machine used, where an Ingenia 1.5T scanner was exclusively used. To prove the formulae to be independent of the machine, mapping sequence and parameters used, still more studies need to be done.

The rationale for adding post-contrast values was, that adding more data to the calculation could improve the formula. The post-contrast T1 relaxation time of the blood pool was chosen, because post-contrast mapping is crucial in the calculation of ECV and therefore adding it is simple. Post-contrast values could be possibly affected by age and other factors, but cohorts with different diagnosis were enrolled and the results showed improvement of the formulae regardless of age, gender and patient history.

Using contrast agent brings several dependencies affecting the blood T1 relaxation times—for example type of the agent used, its pharmacokinetics and dosage. As for the types—in most centres macrocyclic gadolinium agents are being used (same as in this case), reducing the possible bias. Pharmacokinetics is still a discussed topic—but as for example Czock et al. [23] stated, gadolinium based agents are supposed to be distributed rapidly after the administration, and eliminated by the kidneys in a fast initial elimination (half-life approximately 2 h) and followed by a slow elimination phase (half-life approximately 6 days) [23]. Although the time after application is constant (15 min) and the dosage is weight adjusted (0.2 mmol/kg) further reducing the bias, this again advocates towards the need of more multicentre studies.

Control group in this study consists of patients indicated to CMR examination but with negative results. Since patients showed symptoms they can´t be considered as healthy, but since relaxation times from blood pool and not myocardium were used and we find ethically problematic applying contrast agent to healthy volunteers without any clinical outcome from CMR examination, no healthy controls were enrolled in this study. Also, study population did not include any subjects with extreme values of Htc, such as anaemic patients or patients with polycythaemia vera.

## Conclusion

After a careful statistical analysis, the linear and reciprocal formulae for non-invasive calculating of Htc were created, at first using only native blood pool values, afterwards using both native and post contrast values. With assessed synthetic Htc values ECV were quantified and compared with values calculated using other formulae and laboratory methods. The synthetic Htc and ECV quantification were best explained by a model using reciprocals of native and post contrast values: “Htc_est_ = − 0.047 + (779/(T_1-BN_)) − (11.36/T_1-BP_)”; r^2^ = 0.49. Adding post-contrast values to the formula proved to be statistically significant. A residual analysis showed a normal distribution of residuals, confirming the adequacy of our formulae. The formulae statistically proved to apply to the whole study population, regardless of the primary diagnosis or age.

In terms of ECV itself, the models based on our own data had negligible bias close to 0, while using Treibel’s formulae, there was a significant bias in the case of MOLLI-based empirical formulae that rendered significantly lower results with around 5% bias in both cases, but significantly lower bias in the case of ShMOLLi-based formula. This further implies statement Treibel made in his study [17], that potentially a local calibration in every single centre for synthetic Htc and ECV calculations are required.

## Data Availability

The datasets used during the current study are available from the corresponding author on reasonable request.
